# Optimization of Drug Therapy for Heart Failure With Reduced Ejection Fraction Based on Gender

**DOI:** 10.1007/s11897-022-00583-w

**Published:** 2022-10-05

**Authors:** Massimo Iacoviello, Rosanna Pugliese, Michele Correale, Natale Daniele Brunetti

**Affiliations:** 1 Department of Medical and Surgical Sciences, University of Foggia, Viale Luigi Pinto 1, Foggia, Italy; 2 Cardiology Unit, University Polyclinic Hospital of Foggia, Foggia, Italy

**Keywords:** Heart failure, Therapy, Gender, Body surface area

## Abstract

**Purpose of Review:**

Over the last decades, several classes of drugs have been introduced for the treatment of patients with heart failure with reduced ejection fraction (HFrEF). Their use has been supported by randomized controlled trials that have demonstrated improved patient outcomes. However, these trials enrolled a small number of female patients and sometimes have reported gender-related differences regarding the efficacy of the treatments. The aim of this review is to revise the available data about the influence of gender on the optimal treatment and drug dose in patients with HFrEF.

**Recent Findings:**

Several gender-related differences in terms of pharmacokinetic and pharmacodynamic characteristics of the drugs have been described. These characteristics could be responsible for a different response and tolerability in men and women also when current recommended treatment of HFrEF is considered. Some studies have shown that, in women, lower doses of beta-blockers and inhibitors of renin angiotensin aldosterone system could be equally effective than higher doses in men, whereas sacubitril/valsartan could exert its favorable effect at greater values of left ventricular ejection fraction.

**Summary:**

Although there is evidence about differences in the response to treatment of HFrEF in men and women, this has not been sufficient for differentiating current recommended therapy. Further studies should better clarify if the treatment of HFrEF should be based also on the patients’ gender.

## Introduction

Over the last decades, several randomized controlled trials (RCTs) have demonstrated the efficacy of several classes of drugs in improving the prognosis of patients with heart failure with reduced ejection fraction (HFrEF) [1–18]. Consequently, European and American guidelines have recommended these drugs for the treatment of patients with chronic heart failure (CHF) [19, 20].

However, all trials that evaluated the efficacy of the current therapeutic approaches for CHF had a small number of female patients [21.••, 22]. This issue is even more relevant considering that female patients are naturally different from male patients and that these differences may affect the pharmacokinetics and pharmacodynamics of drugs [23.••, 24.••].

These differences could be related also to the body composition, which represents one of the other relevant aspects influencing the effectiveness of any therapeutic approach [25]. This is due to the influence of the body’s composition on drug distribution and clearance as well as on hemodynamic properties such as the clearance of natriuretic peptides [25, 26].

Hence, the aim of this review is to revise the available data about the influence of gender on the optimal drug dose for heart failure patients.

## Pharmacokinetic and Pharmacodynamic Differences According to Gender and Body Composition

**Fig. 1:**
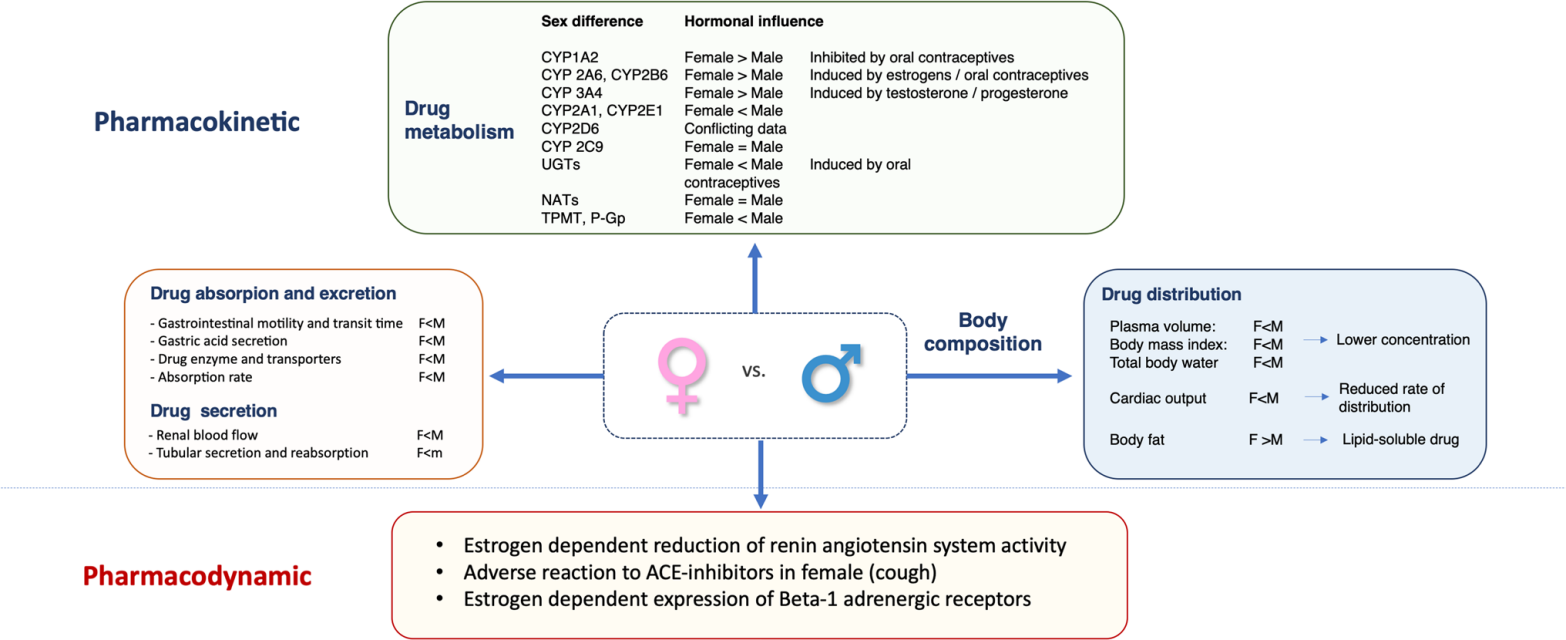
Differences in pharmacokinetic and pharmacodynamic in male and female are summarized. CYP, cytochrome P; F, female; M, male

Several gender-related differences have been demonstrated to affect a drug’s pharmacokinetics. In women, the higher plasma concentration of drugs is due to differences in absorption (slower gastrointestinal motility and transit time, lower absorption, and less drug enzymes and transporters), metabolism, distribution, and excretion (lower renal blood flow and glomerular filtration rate, slower clearance, and longer half-life) [23.••, 24.••]. Figure 1 summarizes the pharmacokinetic and pharmacodynamic differences according with the gender.

### Gender-Related Differences Affecting Drug Metabolism

Gender-related differences in phase I drug metabolism are evident in cytochrome P (CYP) activity. In women, CYP450 activity is lower [23.••, 24.••], which can be attributed to the fact that endogenous hormones, including estrogens and progestins, are also metabolized by this enzyme [23.••, 24.••]. Additionally, activity of CYP450 isoenzymes as well as those of CYP1A2 and CYP2E1 are higher in men, whereas CTP2A6, CYP2B6, and CYP3A4 have higher activity in women [23.••]. There are conflicting results about the activity of CYP2D6 in men and women [27]. In phase II drug metabolism, methyltransferase and sulfotransferase activity is higher in men, whereas uridine diphosphate glucuronosyltransferase and N-acetyl-transferase activity is higher in women [23.••, 24.••].

### Gender, Body Composition, and Pharmacokinetics

Other relevant gender-related differences that may affect drug pharmacokinetics are body surface area, body weight, and body composition [23.••, 25]. Generally, women weigh less and are shorter but have a higher proportion of body fat than men, which result in slower drug clearance due to a lower glomerular and hepatic filtration rate, leading to higher drug plasma concentrations. On the other hand, the increased adipose tissue can influence drug distribution, particularly of lipophilic drugs. Hydrophilic drugs generally have a smaller volume of distribution in adipose tissue and a higher plasma concentration, whereas lipophilic drugs have greater distribution in adipose tissue, which could result in lower plasma concentrations when there is increased adipose tissue [25]. Another aspect relative to body composition is related to drug clearance [22, 23.••, 25]. Hepatic metabolism, which is dependent on cardiac output and liver blood flow, is lower in women, whereas gender differences in renal excretion are related to body weight.

### Gender-Related Pharmacodynamic Differences in Heart Failure Therapy

Aside from drug pharmacokinetics, drug pharmacodynamics could be also influenced by gender-related differences related to hormonal and non-hormonal factors.

Estrogens inhibit the renin-angiotensin system. After an initial increase in the angiotensin II (ATII) plasma levels, angiotensin converting enzyme (ACE) and renin activity decrease due to negative feedback, resulting in a reduced expression of type-1 ATII receptors with a net inhibitory effect [28–30]. Moreover, after menopause, lower ACE activity is improved by hormone replacement therapy [31].

Differences have also been observed when sympathetic activity is considered. The expression of beta-1 adrenergic receptors could be influenced by estrogen [32–34], which can explain the greater reduction of arterial blood pressure and heart rate in women than in men despite receiving similar doses of the same drug [35, 36].

## The Evidence in RCT According to Gender

**Fig. 2:**
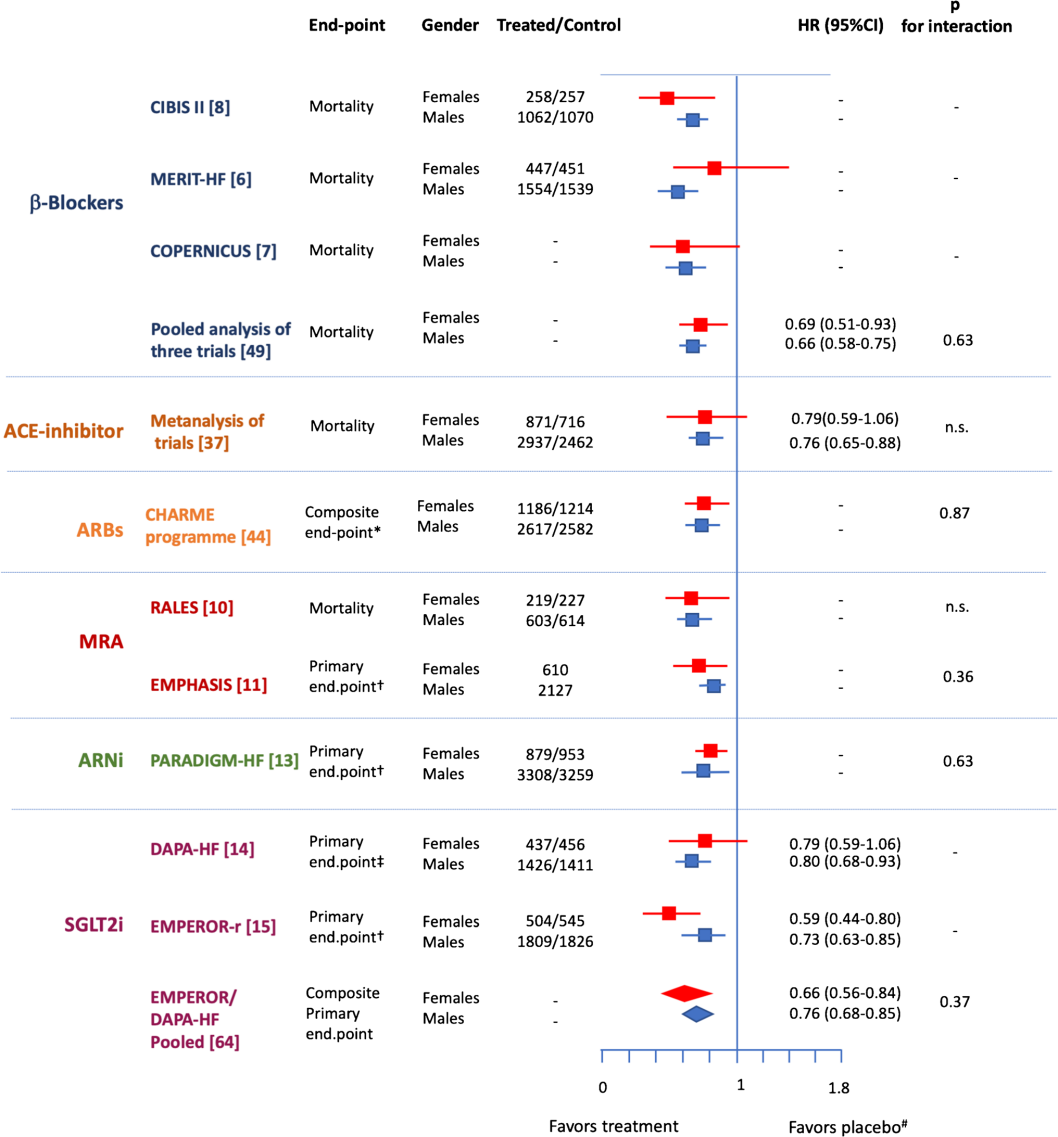
Analyses for male and female subgroups in the main randomized controlled trials evaluating the efficacy of heart failure drugs. ACE, angiotensin I converting enzyme; ARBs, angiotensin II receptor blockers; ARNi, angiotensin II receptor neprilysin inhibitors; CI, confidence interval; HR, hazard ratio; MRA, mineralocorticoid receptor antagonist; n.s., not significant interaction; SGLT2i, inhibitors of type 2 renal sodium-glucose co-transporter. *Composite end-point: Cardiovascular death or first admission for heart failure; †death from cardiovascular causes or hospitalization for heart failure; ‡composite of worsening heart failure (hospitalization or an urgent visit resulting in intravenous therapy for heart failure) or cardiovascular death; ^#^for PARADIGM-HF sacubitril/valsartan vs. enalapril

The current evidence regarding gender-related differences in HF therapy is influenced by the under-enrollment of female patients. The under-enrollment of female patients is evident in most of the RCTs that have demonstrated benefits of HF drugs [21.••, 22]. In a systematic review of RCTs involving 183,097 patients with HFrEF, only 25.5% were female [21.••]. Moreover, female patients were under-enrolled in 71.6% of the RCTs; this proportion did not increase significantly between 2000 and 2019. Furthermore, many trials do not separately report gender-related risk factors and comorbidities as well as the adverse effects of drugs according to gender. Aside from the under-enrollment of female patients, another concern is the gender-related benefits of drugs for patients with heart failure. Figure 2 summarizes the subgroup analyses of the RCTs which are described below and current recommended HFrEF therapy is based on.

### Renin–Angiotensin–Aldosterone System Blockade

Two meta-analyses that evaluated ACE inhibitors in patients with CHF showed that men benefited more from ACE inhibitor therapy than women [37, 38], with mortality and/or hospitalization reduced by 37% in men vs. 22% in women [37]. However, after a myocardial infarction complicated by left ventricular dysfunction, no gender-related differences were observed in a meta-analysis of most of the available trials [39]. Regarding ACE inhibitors, women have a greater incidence of cough than men [40], whereas no gender-related difference was observed when angioedema was considered [41].

Additionally, the ELITE II, Val-HeFT, and CHARM trials demonstrated that there were no gender-related differences for ARBs [42–44].

Spironolactone and eplerenone have demonstrated their ability in reducing mortality among patients with HFrEF and affect both genders equally [10, 11]. Moreover, the Randomized ALdactone Evaluation Study and Eplerenone Post-Acute Myocardial Infarction Heart Failure Efficacy and Survival Study, wherein there was a low percentage of female patients (27% and 28%, respectively), revealed that mineralocorticoid receptor antagonists (MRA) had similar benefits in both genders [45]. A recent meta-analysis [46], including TOPCAT, confirmed that spironolactone had similar benefits for both male and female patients with heart failure with preserved ejection fraction (HFpEF).

In this analysis, analogous to a real-world study [47], women were different from men because they were older and had a higher body mass index, poorer renal function, and fewer comorbidities except for essential hypertension. However, MRA treatment had a similar effect in both men and women in terms of cardiovascular death and hospitalization due to HF, cardiovascular death alone, and all-cause death regardless of the possible confounding factors. Analogously, MRA-related hyperkalemia as well as worsening renal function did not vary by gender.

### Beta-Blockers

Some data suggest that the pharmacokinetic and pharmacodynamic differences in men and women could be related to the different efficacies of metoprolol and carvedilol as demonstrated by the Metoprolol Controlled Release/Extended Release Randomized Intervention Trial in Chronic Heart Failure [6, 48] and Carvedilol Prospective Randomized Cumulative Survival [4]. On the other hand, CIBIS-II did not demonstrate differences between male and female patients [7, 49]. These results could be related to the metabolism of carvedilol and metoprolol, which, differently from that of the bisoprolol, is CYP2D6 dependent (Table 1) [50]. However, as above mentioned, the results of the studies evaluating CYP2D6 in men and women are conflicting [27]. The other possible explanation is related to the small proportion of enrolled female patients in these trials. This hypothesis is strengthened by a meta-analysis that pooled the results of these trials and demonstrated a similar effect of these drugs in reducing mortality in women [49].

**Table 1:** Main pharmacokinetic and pharmacodynamic characteristics of disease modifiers’ drugs from https://www.ema.europa.eu/en/documents/product-information/. *ACE* angiotensin converting enzyme, *ARBs* angiotensin II receptor blockers, *ARNi* angiotensin II receptor neprilysin inhibitors, *CYP* cytochrome P450, *MRA* mineralocorticoid receptor antagonists, *SGLT2i* inhibitors of sodium-glucose cotransporter

Drugs	Metabolism	Excretion	Gender related pharmacokinetic difference	Gender related pharmacodynamic difference
ACE-inhibitors				
Enalapril	Hydrolysis to Enalaprilat	Renal	Yes	In female: - Estrogen mediated RAS inhibition; - Greater sensitivity to lower doses; - Increased incidence of cough
Lisinopril	No metabolism	Renal	No	
Ramipril	Ramiprilat Glucuronidation	Renal	Yes	
Captopril	Sulfuration (40/50%)	Renal	No	
Trandolapril	Trandolaprilat	Renal (33%) Fecal (66%)	Yes	
ARBs				
Candesartan	2C9 (weak)	Renal ( +)/hepatic	No	No gender differences described
Losartan	CYP2C, 3A4, 1A2	Renal/hepatic ( +)	No	
Valsartan	2C9 (weak)	Renal/hepatic ( +)	No	
ARNi				
Sacubitril/(Valsartan)	Esterases (active metabolite sacubitrilat)	Renal	No	No gender differences described
MRA				
Spironolactone	Hepatic, active metabolites (including canrenone)	Renal	No	No gender differences described
Canrenoate	Converted to canrenone	Renal	No	
Canrenone	-	Renal	No	
Eplerenone	Cyp 3A4 (inactive metabolites)	Renal	No	
Beta-blockers				
Carvedilol	Hepatic by first-pass metabolism CYPs 2D6 and 2C9	Non-renal	Yes	In female: - Different expression of beta-1 adrenergic receptor; - Greater sensitivity to lower doses;
Bisoprolol	Hepatic	Renal (50%)	No	
Nebivolol	Hepatic first-pass metabolism by CYPs2D6 and 2C9	Renal (60%)	Yes	
Succinate metoprolol	Hepatic by first-pass metabolism CYP2D6	Renal	Yes	
SGLT2i				
Dapagliflozin	Glucuronidation	Renal (75%)	No	No gender differences described
Empagliflozin	Glucuronidation	Renal (54%)	No	
Sotagliflozin	Glucuronidation	Renal (57%) Fecal (37%)	No	

### Sacubitril/Valsartan

Possible gender-related differences have been suggested in the response to therapy with sacubitril/valsartan, which are recommend for patients with HFrEF [13]. Sacubitril and valsartan inhibit ATII and neprilysin, respectively. Neprilysin is an endothelial endopeptidase involved in the degradation of natriuretic peptides that counteracts the overactivation of RAAS and sympathetic nervous system by inducing natriuresis and diuresis as well as exerts an antifibrotic effect at the cardiac level [51]. Sacubitril/valsartan also showed a strong effect on cardiac remodeling, which is related to the reduction of serum levels of natriuretic peptides [52, 53]. Notably, the degree of reverse remodeling and the reduction of NT-proBNP levels were greater in women with HFrEF after sacubitril/valsartan therapy [54]. The possibility that sacubitril/valsartan exert different effects in male and female patients has been strengthened by the results of PARAGON-HF [55, 56.••]. PARAGON-HF compared treatment between sacubitril/valsartan with valsartan alone in patients with CHF and a left ventricular ejection fraction (LVEF) ≥ 45%, evidence of structural heart disease (left atrial enlargement or left ventricular hypertrophy), New York Heart Association classes II–IV, and elevated levels of natriuretic peptides. The results of PARAGON-HF demonstrated that sacubitril/valsartan reduced hospitalizations due to HF and cardiovascular mortality, albeit without statistical significance (rate ratio: 0.87; 95% confidence interval [CI]: 0.75–1.01; *P* = 0.06) [56.••]. Among the pre-specified sub-groups, heterogeneity was observed in the subgroup of female patients who benefited more from sacubitril/valsartan therapy.

The differential benefits of sacubitril/valsartan in female patients have also been suggested by an analysis that combined data from PARADIGM-HF and PARAGON-HF [57]. When the population of the two studies was combined, a benefit was evident in patients with an LVEF < 55%. However, in female patients, the benefit was greater in patients with higher LVEF values. The explanation for this gender-based relationship between LVEF and the beneficial effects of sacubitril/valsartan is unclear. One is that systolic dysfunction in female patients is already present at a higher LVEF [58–60]. The varied response to neurohormonal modulation at a greater LVEF among women is further supported by the results of the TOPCAT and CHARM trials on the effects of spironolactone and candesartan, respectively [61].

Another possible explanation may be attributed to the differences in the natriuretic peptide system between male and female patients, particularly in those with HFrEF or HFpEF. Among these patients, women had lower levels of natriuretic peptides despite a similar severity of HF. This is probably due to the increased clearance of natriuretic peptides related to greater visceral obesity as well as to the reduced levels of natriuretic peptides after menopause, which lead to a relative insufficiency of natriuretic peptides in women that may be improved by sacubitril/valsartan [61].

### Type 2 Sodiun-Glucose Cotrasporter Inhibitors

A possible gender-related influence in the efficacy of SGLT2i was hypothesized in diabetic patients [62, 63]. In DAPA-HF [14] and in EMPEROR-reduced [15], the proportion of female patients was 19.6 and 22.8%, respectively. Interestingly, in a meta-analysis of the two trials [64], a similar reduction of the primary endpoint was observed in men (hazard ratio [HR]: 0.76; 95% CI: 0.68–0.85) and in women (HR: 0.68; 95% CI: 0.56–0.84).

### Digoxin

The first evidence of possible gender-related differences was derived from the analysis of the DIG trial, which showed a significant interaction between digoxin administration and events among male and female patients [65]. The general results of the trial demonstrated that digoxin was associated with increased mortality in female patients. Moreover, in women, a smaller digoxin-associated reduction in the rate of hospitalization due to heart failure was observed. Interestingly, the mean daily dose of digoxin was not different between men and women (*p* = 0.28), but the median serum digoxin level at 1 month was slightly higher in a subgroup of women than in a subgroup of men (*p* = 0.007). This suggests the possibility of differences in pharmacokinetics between men and women that could be attributed to an interaction between hormone replacement therapy and digoxin. Progestin may increase serum digoxin levels by inhibiting P glycoprotein, thus reducing the renal excretion of digoxin through the renal tubules [65]. This interaction is supported by the Heart and Estrogen/Progestin Replacement Study, wherein it was observed that the interaction between digoxin and hormone replacement therapy was associated with a higher rate of cardiovascular events [66].

## Differences in the Dose/Effectiveness of HFrEF Treatment According to Gender

Although the classes of drugs currently recommended for the treatment of HFrEF show similar efficacy regardless of gender, beneficial effects were noted depending on the doses of drugs.

The ATLAS study [67] was a randomized controlled trial aimed to compare the effects of a high dose (32.5 to 35 mg daily) with those of a low dose (2.5 to 5.0 mg daily). Among more than 3000 patients with heart failure, LVEF ≤ 30% and NYHA classes II–IV only 26% in low-dose group and 20% in the high-dose group were female. The trial did not show a significant effect of high dose on death, but a significant lower occurrence of hospitalizations related to heart failure was observed. However, when gender was considered in the subgroup analysis, a trend toward a greater beneficial effect in men than in women was observed.

In the HEAAL study [68], the effects of a high dose of losartan (150 mg/daily) versus a low dose (50 mg/daily) were compared in a group of patients with HFrEF (LVEF at the enrollment ≤ 40%). No differences were found among groups in the occurrence of the combined end-point death or admission for heart failure. Like most of the other RCTs in heart failure, HEAAL study was also characterized by an underenrollment of female patients (30% in the high dose group and 29% in low dose group). Interestingly, in the subgroup analysis, women showed a less beneficial effect form high dose of losartan than men, with a *p* for interaction near to the statistical significance (*p*: 0.10).

More recently, the possibility that lower doses of HFrEF diseases modifier drugs can be effective in female like the higher in males has been further supported by a post hoc analysis of BIOSTAT-CHF [69.••], which was a prospective study involving centers of 11 European countries that was aimed to evaluate the initiation and up-titration of ACE inhibitors or ATII receptor blockers and beta-blockers among patients with HFrEF. Among the 1710 patients with HFrEF, 30.7% were female and were older; there was no difference in BMI between male and female patients. The percentage of male and female patients in whom the target dose of ACE inhibitors or ATII receptor blockers and beta-blockers was reached was similar. However, the benefit observed in female patients in terms of mortality reduction was significant at doses lower than those in men. The differences between male and female patients remained significant after correcting for covariates, including age and body surface area. Interestingly, despite differences in baseline characteristics and ethnicity, similar results were observed among patients enrolled in the ASIAN-HF registry.

## Conclusions

Studies have demonstrated gender-related differences in the pharmacokinetic and pharmacodynamic properties of drugs currently recommended for patients with HFrEF, which is related to the difference in body composition between men and women. Moreover, although most of the RCTs have demonstrated that drugs have similar efficacy in male and female patients, some evidence suggests the possibility that a lower dosage could be as effective in women.

Despite this evidence, current European and American guidelines [19, 20] do not recommend personalized treatment based on gender and body composition in HFrEF therapies. Further studies should better clarify if HFrEF treatment should be tailored based on gender and body composition.

## Abbreviations

ACE: Angiotensin-converting enzyme
ATII: Angiotensin II
CHF: Chronic heart failure
CYP: Cytochrome P
HFpEF: Heart failure with preserved ejection fraction
HFrEF: Heart failure with reduced ejection fraction
LVEF: Left ventricular ejection fraction
MRA: Mineralocorticoid receptor antagonists
RAAS: Renin-angiotensin-aldosterone system
RCTs: Randomized controlled trials
